# Group-Based Suicide Safety Planning for Veterans with High Suicide Risk Via In-Person and Telehealth Delivery

**DOI:** 10.1007/s41347-026-00719-y

**Published:** 2026-09-18

**Authors:** Sofie Glatt, Gregory K. Brown, Shari Jager-Hyman, Michael E. Thase, Maureen Monahan, Sheila O’Brien, Alison Krauss, Hanga C. Galfalvy, Sarah R. Sullivan, Emilia Fonseca, Marianne Goodman

**Affiliations:** 1 VISN 2 Mental Illness Research, Education, and Clinical Center (MIRECC), James J. Peters Veterans Affairs Medical Center, Bronx, NY USA; 2 Department of Psychiatry, University of Pennsylvania Perelman School of Medicine, Philadelphia, PA USA; 3 Corporal Michael J. Crescenz VA Medical Center, Philadelphia, PA USA; 4 Department of Psychiatry, Vagelos College of Physicians and Surgeons, Columbia University, New York, NY USA; 5 New York State Psychiatric Institute, New York, NY USA; 6 VISN 17 Center of Excellence for Research on Returning War Veterans, Central Texas Veterans Health Care System, Waco, TX USA; 7 Department of Psychology, Hunter College and the Graduate Center, City University of New York, New York, NY USA; 8 Department of Psychiatry, Icahn School of Medicine at Mount Sinai, New York, NY USA

**Keywords:** Suicide, Group Treatment, Telehealth, Veteran, Suicide-Coping

## Abstract

**Objective:**

Suicide-specific group psychotherapy is a novel approach to suicide prevention with promising supporting data. However, limited research examines telehealth delivery for suicide-specific interventions. This study described treatment engagement and suicide-related coping (SRC) trajectories among Veterans receiving a group suicide safety planning intervention before and after a transition from in-person to telehealth delivery.

**Methods:**

Project Life Force (PLF) is a group suicide safety planning intervention evaluated within a randomized clinical trial. During the COVID-19 pandemic, PLF transitioned from in-person to telehealth, creating naturalistic pre-pandemic in-person and pandemic-era telehealth cohorts. This report focused on participants randomized to PLF and described treatment initiation, engagement, completion, and SRC trajectories across cohorts.

**Findings:**

: Baseline suicidal ideation and SRC were higher in the pre-pandemic in-person cohort than the pandemic-era telehealth cohort. The telehealth cohort showed higher rates of treatment initiation descriptively than the in-person cohort, while treatment engagement and completion among treatment initiators were more similar across delivery periods. Both cohorts showed improvement in SRC that was sustained over time.

**Conclusions:**

Elevated treatment initiation occurred with virtual delivery of PLF. Both pre-pandemic in-person and pandemic-era telehealth cohorts showed similar patterns of group engagement as well as development and sustainment of SRC over time. As telehealth can reduce barriers to care, these data lend support to the promise of virtual, group-based, suicide-related interventions. Since treatment modality was determined by the pandemic and not randomized, delivery context, cohort characteristics, and pandemic-related factors may have impacted observed patterns. Randomized studies comparing delivery modalities should clarify potential treatment modality differences.

**Supplementary Information:**

The online version contains supplementary material available at https://doi.org/10.1007/s41347-026-00719-y.

## Introduction

Suicide remains a major public health concern in the United States and is the 11th leading cause of death. Veterans are disproportionately affected, with suicide rates more than twice those of civilians after adjusting for age and sex (U.S. Department of Veterans Affairs [VA], Office of Suicide Prevention, 2024). Despite sustained national suicide prevention efforts, over 6,400 veterans died by suicide in 2022 (VA, Office of Suicide Prevention, 2024). In response, the Department of Veterans Affairs (VA) and other federal agencies have launched initiatives to facilitate suicide risk detection, prevention, and treatment.

Group-based suicide-specific psychotherapy is a promising but underdeveloped approach to reducing suicide risk. Compared with individual therapy, group interventions can reduce social isolation and foster community and belongingness, which are important protective factors against suicide (Joiner et al., 2009). They also create opportunities for reciprocal support by allowing participants to both give and receive help (Bowersox et al., 2021), which may align particularly well with Veterans’ team-oriented military culture and experiences (Whitesell & Owens, 2012). Additionally, group formats are cost-effective and may increase treatment capacity within healthcare systems (Raciborski et al., 2023). Despite these advantages, relatively few group interventions for suicide have been developed and tested. A recent PRISMA scoping review identified only ten suicide-focused group interventions, including seven open-label trials and three randomized clinical trials [RCTs] (Sullivan et al., 2021). While this reflects a limited evidence base, the studies generally reported improvements in suicide-related outcomes (Sullivan et al., 2021).

One intervention identified in the review, Project Life Force (PLF) (Goodman et al., 2020, 2026), is a manualized 10–12 session group treatment designed to strengthen Veterans’ suicide safety plans (SSPs) (Stanley & Brown, 2012). PLF integrates elements of Dialectical Behavior Therapy (DBT) and psychoeducation to promote emotion regulation, suicide-related coping (SRC), and perceived belonging. PLF addresses all six steps of the SSP and includes modules on physical well-being, communicating SSPs with family/significant others, and reasons for living (Goodman et al., 2020, 2021). Each week, Veterans refine their SSP while learning skills intended to strengthen the plan, with the primary goal of maximizing SRC. Unlike many skill-based groups, PLF directly discusses suicidal thoughts and behaviors to emphasize application of coping skills and risk management. PLF aims to increase self-efficacy and reduce suicide risk through shared experiences and mutual support.

The COVID-19 pandemic necessitated a rapid transition from in-person to telehealth services for healthcare systems and psychosocial interventions, including PLF (Betthauser e et al., 2025; Carper et al., 2022; Landes et al., 2026; Puspitasari et al., 2021; Rosen et al., 2021; Stepanova et al., 2024). Telehealth interventions have demonstrated feasibility, patient satisfaction, and comparable effectiveness to in-person treatment across a range of behavioral and psychological conditions (Bellanti et al. 2022; Lancaster et al. 2025; McClellan et al. 2022; Swartz et al. 2023a). In addition, telehealth may facilitate treatment initiation and uptake by easing logistical barriers and increasing accessibility (Swartz et al. 2023b; Yellowlees and Shore 2018). However, investigations of how group treatment processes occur in different modalities are limited, particularly for suicide-specific treatments. Existing work suggests that telehealth group therapy might alter key group therapeutic components but promote accessibility and yield comparable outcomes to in-person group therapy (Foy et al., 2010; Gentry et al., 2018; Kneeland et al., 2021; Lopez et al., 2020). For PLF specifically, early qualitative and economic evaluations during the pandemic indicate favorable perceptions, maintained therapeutic processes, improvements in suicide-related symptoms, and reduced delivery costs (Patel et al., 2023; Raciborski et al., 2023).

The transition from in-person to telehealth delivery created a naturalistic opportunity to explore PLF cohorts recruited before the pandemic, when treatment was delivered in-person, and during the pandemic (i.e., after its onset), when treatment was delivered via telehealth. Because the treatment modality shift was prompted by the pandemic, which disrupted daily life and experiences, our aim was to describe, rather than formally compare, patterns across the pre-pandemic in-person and post-pandemic telehealth cohorts (Rabner et al., 2025). In this report, we describe baseline clinical characteristics, treatment engagement patterns, and trajectories of SRC across delivery periods.

## Methods

### RCT Design

Of 294 high-risk suicidal Veterans assessed for eligibility, 207 were randomized to Project Life Force (PLF) in addition to Treatment as Usual (TAU) or TAU alone between March 2018 and February 2024 at four VA Medical Centers (VAMCs): James J. Peters VAMC (JJPVA; *n* = 129), Corporal Michael J. Crescenz VAMC (CMJCVA; *n* = 59), Central Texas Veterans Health Care System (CTVCS; *n* = 17), and Northport VAMC (*n* = 2). The present report focuses on participants randomized to PLF (*n* = 101). Study procedures were approved by each VAMC’s Institutional Review Board and Research and Development Office. The RCT was registered at ClinicalTrials.gov (NCT03653637; registered on 2018-08−31).

## RCT Participant Recruitment and Procedures

Participants were recruited from (1) inpatient psychiatric units after a suicide-related admission, (2) VA high-risk lists managed by Suicide Prevention Coordinators, and (3) outpatient mental health clinics for Veterans with a suicide attempt in the past year or reported active suicidal ideation (SI) with at least some intent within the past month. Full inclusion/exclusion criteria and details are outlined in the protocol paper (Goodman et al., 2020).

The control condition in the parent RCT was TAU, operationalized as the current standard of care for suicidal individuals discharged from inpatient units or identified as high-risk. The primary methodological difference between conditions was that participants randomized to PLF also had the option to attend PLF group sessions to augment their SSPs. Since PLF was structured to maximize treatment availability and accessibility, participants randomized to PLF could begin attending sessions immediately after completing baseline assessment rather than waiting for the next intervention cycle.

## Assessments

The full RCT assessment battery is described in the study registration. Primary and secondary outcome measures were administered at baseline and all follow-up assessments (months 3, 6, and 12 post-baseline). This report focuses on treatment engagement and SRC. Other variables include lifetime suicide attempt and baseline SI, depression symptom severity, hopelessness, and attitudes toward seeking professional psychological help.

### Treatment Engagement

Session attendance was measured by counting the number of PLF sessions attended. “Initial engagement” was defined as attending at least one PLF session. “PLF treatment engagement” was defined as attendance ≥ 6 sessions, corresponding to the number of sessions reviewing SSP steps, and “PLF treatment completion” required attendance of the full ten-session cycle.

### Columbia Suicide Severity Rating Scale

**(**C**-**SSRS; Posner et al., 2011). The C-SSRS is a reliable semi-structured clinical interview that assesses lifetime and recent suicide attempts (actual, aborted, and interrupted). The current report examined lifetime (actual) suicide attempts at baseline (see Data Analytic Strategy). Disagreements in attempt classification were resolved by consensus among raters.

### Suicide Related Coping Scale

(SRCS; Stanley et al., 2017). The SRCS has 17 items assessing knowledge and confidence in using internal coping strategies and external supports to regulate suicidal thoughts and urges. Items are rated on a 5-point scale (*Strongly disagree* (0) – *Strongly agree* (4)). Higher scores indicate better SRC. All items are summed for a general SRC score, and the first 14 items are summed for “external coping” and “internal coping” subscales (7 items each). The SRCS has acceptable internal and external validity and weak-to-moderate correlations with SI (Stanley et al., 2017). Internal consistency was good at baseline (α > 0.80 in the Intent-to-Treat (ITT) and treatment-initiator samples) and all follow-ups (all α > 0.80 in the treatment-initiator sample).

### Beck Scale for Suicide Ideation

(SSI; Beck et al., 1979). The SSI is a 19-item self-report measure designed to quantify suicidal ideation (SI). The first five items function as a screener; participants scoring > 0 on items four and five completed the full scale. Higher scores indicate more severe suicidal symptoms. Internal consistency was good at baseline (α > 0.80 in the ITT and treatment-initiator samples, calculated for participants that completed the full scale).

### Beck Depression Inventory-II

(BDI-II; Beck et al., 1996). The BDI-II is a 21-item measure of depression symptom severity over the past two weeks. Items are rated on a scale from 0 to 3 in increasing severity and are summed for a total score (range 0–63). Higher scores indicate more severe depression symptom severity. Internal consistency was good at baseline (α > 0.80 in the ITT and treatment-initiator samples).

### Beck Hopelessness Scale

(BHS; Beck & Steer, 1988*).* The BHS includes 20 true/false items assessing pessimistic and optimistic attitudes about the future. Items were recoded (optimistic beliefs = 0 and pessimistic beliefs = 1) and summed for a total score (range 0–20). Higher scores indicate more severe hopelessness. Internal consistency was good at baseline (α > 0.80 in the ITT and treatment-initiator samples).

### Attitudes Towards Seeking Professional Psychological Help-Short Form

(ATSPPH-SF; Fischer & Turner, 1970; Fischer & Farina, 1995). The ATSPPH-SF (Fischer & Farina, 1995) was adapted from the original ATSPPH (Fischer & Turner, 1970) to measures attitudes toward seeking professional help for psychological disturbance with 10 items. Items rated on a 4-point scale ranging from 0 (disagree) to 3 (agree). Higher scores indicate more positive attitudes toward seeking psychological help. Internal consistency was acceptable at baseline (α ≥ 0.70 in the ITT and treatment-initiator sample).

## Description of the PLF-only Sample

Of the 101 Veterans randomized to PLF, 70 initiated treatment (≥ 1 session). Twenty-two (31.4%) received the intervention in-person, 44 (62.9%) received it entirely via telehealth, and four (5.7%) received it with a hybrid of both modalities. Across sites, 50 participants (71.4%) received treatment from the JJPVA, 15 (21.4%) from CMJCVA, and five (7.1%) from CTVCS. At JJPVA, 16 (32%) were treated in-person, and 34 (68%) were treated via telehealth (including the four hybrid cases). At CMJCVA, six (40%) received in-person and nine (60%) received telehealth, and all CTVCS participants received telehealth. The four participants who received a hybrid mix of modalities were excluded from the treatment-initiator sample for longitudinal descriptions (see Supplementary Fig. 1), and an additional two participants were excluded due to missing baseline scores.

The sample randomized to PLF (*N* = 101) had descriptively fewer White Veterans (44.6% vs. 74%) and a higher proportion of Black Veterans (26.7% vs. 12.6%) relative to national Veteran averages (Pew Research Center, 2023). Rates of women Veterans were comparable to the national average (15.8% vs. 11%), and the average age of the sample was younger (46 vs. 62). Rates of some college education or above were similar. For participant demographic information, see Table 1.

**Table 1:** Participant Demographic Information for the Total Sample and by Treatment Modality

Category	Total initiated	Initiated telehealth	Initiated in-person	Total assigned	Assigned in-person	Assigned telehealth
	*N* = 70	*N* = 44	*N* = 26	*N* = 101	*N* = 46	*N* = 55
Age (mean [SD])	45.04 (14.34)	44 (14.04)	46.84 (14.95)	45.88 (13.81)	48.8 (13.96)	43.44 (13.32)
Birth sex						
Female	10 (14.3%)	7 (15.9%)	3 (11.5%)	16 (15.8%)	5 (10.9%)	11 (20%)
Male	58 (82.9%)	36 (81.8%)	22 (84.6%)	81 (80.2%)	38 (82.6%)	43 (78.2%)
Ethnicity						
Hispanic/Latinx	30 (42.9%)	20 (45.5%)	10 (38.5%)	36 (35.6%)	14 (30.4%)	22 (40%)
Non-Hispanic/Latinx	36 (51.4%)	23 (52.3%)	13 (50%)	59 (58.4%)	27 (58.7%)	32 (58.2%)
Race						
Asian	1 (1.4%)	1 (2.3%)	0	2 (2%)	0	2 (3.6%)
Black/African American	19 (27.1%)	9 (20.5%)	10 (38.5%)	27 (26.7%)	17 (37%)	10 (18.2%)
Multiracial	11 (15.7%)	10 (22.7%)	1 (3.8%)	14 (13.9%)	3 (6.5%)	11 (20%)
White	29 (41.4%)	17 (38.6%)	12 (46.2%)	45 (44.6%)	21 (45.7%)	24 (43.6%)
Other	9 (12.9%)	6 (13.6%)	3 (11.5%)	12 (11.9%)	5 (10.9%)	7 (12.7%)
Attendance						
Initiated	24 (34.3%)	14 (31.8%)	10 (38.5%)	24 (23.8%)	10 (21.7%)	14 (25.5%)
Six Sessions	25 (35.7%)	15 (34.1%)	10 (38.5%)	25 (24.8%)	10 (21.7%)	15 (27.3%)
Completer	21 (30%)	15 (34.1%)	6 (23.1%)	21 (20.8%)	6 (13%)	15 (27.3%)
Did not initiate	NA	NA	NA	31 (30.7%)	20 (43.5%)	11 (20%)
Treatment status						
No current psychiatric treatment	1 (1.4%)	0	1 (3.8%)	1 (1%)	1 (2.2%)	0
Outpatient mental health care	40 (57.1%)	30 (68.2%)	10 (38.5%)	48 (47.5%)	11 (23.9%)	37 (67.3%)
Psychiatric hospitalization	18 (25.7%)	8 (18.2%)	10 (38.5%)	40 (39.6%)	29 (63%)	11 (20%)
Partial hospitalization/institutional living	5 (7.1%)	5 (11.4%)	0	6 (5.9%)	0	6 (10.9%)
Education						
High school diploma/GED	18 (25.7%)	15 (34.1%)	3 (11.5%)	24 (23.8%)	9 (19.6%)	15 (27.3%)
Some college/2-year degree	33 (47.1%)	19 (43.2%)	14 (53.8%)	54 (53.5%)	26 (56.5%)	28 (50.9%)
Bachelors degree	13 (18.6%)	8 (18.2%)	5 (19.2%)	15 (14.9%)	6 (13%)	9 (16.4%)
Masters degree	1 (1.4%)	1 (2.3%)	NA	3 (3%)	1 (2.2%)	2 (3.6%)
Employment						
Full-time	9 (12.9%)	8 (18.2%)	1 (3.8%)	16 (15.8%)	5 (10.9%)	11 (20%)
Part-time	3 (4.3%)	2 (4.5%)	1 (3.8%)	4 (4%)	1 (2.2%)	3 (5.5%)
Unemployed	34 (48.6%)	21 (47.7%)	13 (50%)	48 (47.5%)	21 (45.7%)	27 (49.1%)
Student	6 (8.6%)	3 (6.8%)	3 (11.5%)	6 (5.9%)	3 (6.5%)	3 (5.5%)
Retired	11 (15.7%)	8 (18.2%)	3 (11.5%)	20 (19.8%)	11 (23.9%)	9 (16.4%)
Self-employed	1 (1.4%)	1 (2.3%)	0	1 (1%)	0	1 (1.8%)
Marital status						
Married/Cohabiting	18 (25.7%)	11 (25%)	7 (26.9%)	26 (25.7%)	13 (28.3%)	13 (23.6%)
Divorced/Annulled	15 (21.4%)	9 (20.5%)	6 (23.1%)	27 (26.7%)	14 (30.4%)	13 (23.6%)
Separated	14 (20%)	11 (25%)	3 (11.5%)	18 (17.8%)	5 (10.9%)	13 (23.6%)
Never married	19 (27.1%)	12 (27.3%)	7 (26.9%)	25 (24.8%)	11 (23.9%)	14 (25.5%)
Widowed	0	0	0	1 (1%)	0	1 (1.8%)
Pre-military suicidality						
Yes	15 (21.4%)	9 (20.5%)	6 (23.1%)	22 (21.8%)	12 (26.1%)	10 (18.2%)
No	50 (71.4%)	34 (77.3%)	16 (61.5%)	74 (73.3%)	30 (65.2%)	44 (80%)

## Data Analytic Strategy

### Pre-Pandemic and Pandemic-Era Cohorts

To describe pre-pandemic in-person and pandemic-era telehealth cohorts, we summarized baseline characteristics for both treatment modalities, coincident with pandemic onset, for the ITT and treatment-initiator samples. We report descriptive statistics for lifetime suicide attempt history (assessed by the C-SSRS), depression symptom severity (assessed by the BDI-II), hopelessness (BHS), attitudes toward seeking professional psychological help (ATSPPH-SF), suicidal ideation (SSI), and SRC (SRCS-17). These baseline summaries are presented to characterize cohort profiles rather than to support formal group comparisons.

## Treatment Initiation, Engagement, and Completion

Following descriptive characterization of the cohorts recruited prior to the onset of the pandemic (in-person) and during the pandemic (telehealth), we summarized treatment engagement in these cohorts by tabulating rates of treatment initiation (> 0 sessions; ITT sample), engagement (≥ 6 sessions; treatment initiator sample), and completion (≥ 10 sessions; treatment initiator sample). Given the small number of participants in some categories and the non-random association between delivery modality and pandemic period, summaries are presented descriptively to characterize engagement patterns.

## Suicide-Related Coping

To describe SRC trajectories among participants who initiated PLF, descriptive statistics were calculated separately by delivery modality (pre-pandemic in-person and pandemic-era telehealth cohorts). Repeated SRC assessments were summarized across available follow-up time points using means, standard deviations, and ranges. Given the time-dependent, non-random allocation of delivery context, these descriptions were used to summarize SRC trajectories in different delivery periods (pre-pandemic in-person and pandemic-era telehealth).

## Results

### Pre and Post Pandemic Onset Cohorts

Of 101 participants in PLF, 46 were assigned to the in-person treatment modality (pre-pandemic onset) and 55 were assigned to the telehealth treatment modality (during the pandemic). Lifetime suicide attempt history was similar across cohorts in both the ITT and treatment initiator samples. Depression severity, hopelessness, and attitudes toward seeking professional psychological help were broadly similar across cohorts in the ITT and treatment initiator samples (Table 2). SRC was lower in the pandemic-era telehealth cohort than in the pre-pandemic in-person cohort in the treatment-initiator sample (assigned in-person: M = 51.48, SD = 10.70; assigned to telehealth: M = 45.95, SD = 10.75; Table 2). Suicidal ideation was higher in the pre-pandemic in-person cohort than in the pandemic-era telehealth cohort in the ITT (assigned in-person: M = 15.96, SD = 8.69; assigned to telehealth: M = 8.35, SD = 8.39) and treatment initiator (assigned in-person: M = 18.16, SD = 8.24; assigned in telehealth: M = 9.60, SD = 8.49) samples.

**Table 2:** Baseline Clinical Characteristics by Treatment Modality

	*N*	M	SD	Md	MAD	range (min-max)
**Assigned In-Person (*N*** **= 46)**^a^						
BHS	45	10.20	6.58	12.00	8.90	19 (1–20)
ATSPPH-SF	44	21.89	5.14	23.00	5.93	19 (11–30)
BDI-II	45	33.60	13.70	32.00	16.31	53 (6–59)
SSI	45	15.96	8.69	17.00	10.38	32 (0–32)
SRCS-17	45	49.36	11.24	51.00	11.86	47 (20–67)
SRCS-E	45	20.31	4.91	21.00	5.93	21 (7–28)
SRCS-I	45	21.33	4.68	22.00	4.45	20 (8–28)
**Assigned Telehealth (*N*** **= 55)**						
BHS	54	10.04	6.21	10.50	7.413	20 (0–20)
ATSPPH-SF	50	22.74	4.72	23.00	5.9304	16 (14–30)
BDI-II	53	30.70	11.25	32.00	10.3782	48 (3–51)
SSI	51	8.35	8.38	7.00	8.8956	33 (0–33)
SRCS-17	54	47.43	10.42	49.00	8.8956	48 (16–64)
SRCS-E	54	19.26	4.67	20.00	4.4478	27 (0–27)
SRCS-I	54	20.65	4.06	21.00	4.4478	17 (11–28)
**Initiated In-Person (*N*** **= 26)**^a^						
BHS	25	10.88	7.15	12.00	10.38	18 (2–20)
ATSPPH-SF	25	23.16	4.62	23.00	4.45	16 (14–30)
BDI-II	25	33.88	13.24	32.00	16.31	42 (15–57)
SSI	25	18.16	8.24	19.00	11.86	32 (0–32)
SRCS-17	25	51.48	10.70	53.00	10.38	44 (23–67)
SRCS-E	25	21.16	4.75	21.00	5.93	16 (12–28)
SRCS-I	25	22.48	3.95	23.00	4.45	14 (14–28)
**Initiated Telehealth (*N*** **= 44)**						
BHS	43	10.65	6.28	11.00	5.93	20 (0–20)
ATSPPH-SF	43	22.60	4.59	23.00	5.93	16 (14–30)
BDI-II	43	31.23	11.91	32.00	10.38	48 (3–51)
SSI	43	9.60	8.49	10.00	10.38	33 (0–33)
SRCS-17	43	45.95	10.75	48.00	8.90	48 (16–64)
SRCS-E	43	18.56	4.83	19.00	2.97	27 (0–27)
SRCS-I	43	20.33	4.20	21.00	4.45	17 (11–28)

BHS = Beck Hopelessness Scale, ATSPPH-SF = Attitudes Towards Seeking Professional Psychological Help-Short Form, BDI-II = Beck Depression Inventory II, SSI = Beck Scale for Suicidal Ideation, SRC = Suicide-Related Coping, SRCS-17 = SRC Scale, SRCS-E = SRCS External Coping subscale, SRCS-I = SRCS Internal Coping subscale, SD = Standard Deviation, Md = Median, MAD = Mean Absolute Deviation

^**a**^In this table, this category includes individuals who were assigned in-person and transitioned to telehealth (*n* = 4)

### Treatment Initiation, Engagement, and Completion

Twenty-six participants of those assigned to the in-person modality (56.52% of 46) and 44 of those assigned in telehealth modality (80% of 55) initiated treatment. Of those who initiated treatment (*n* = 70), excluding those who transitioned to telehealth (*n* = 4; final *n* = 66), 13 individuals in person (59.09% of 22) and 30 individuals in telehealth (68.18% of 44) engaged in treatment, and 4 individuals in-person (18.18% of 22) and 15 individuals in telehealth (34.09%) cohorts completed treatment. The number of sessions attended for the in-person (*n* = 22) and telehealth (*n* = 44) cohorts were comparable (in-person: *M* = 6.23, *SD* = 3.41, range = 1–11, median = 7.5, IQR = 3–9; telehealth: *M* = 6.91, *SD* = 3.58, range = 1–12, median = 7, IQR = 3.75–10).

### Suicide-Related Coping

**Fig. 1:**
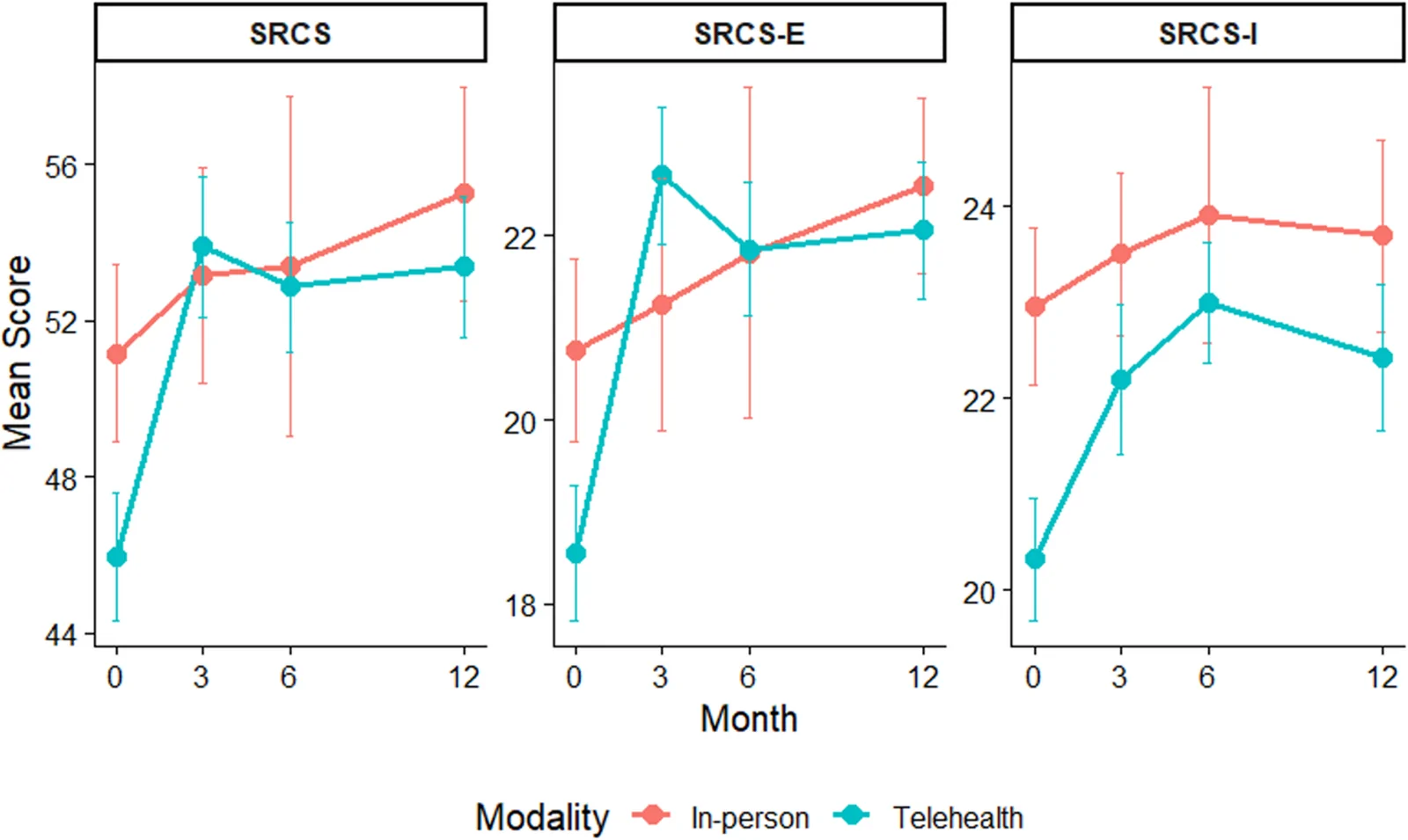
Suicide-related Coping (SRC) Scores Over Time by Treatment Modality

Descriptive statistics of SRC by treatment modality and time for those who initiated treatment (*n* = 70), excluding those who transitioned to telehealth (*n* = 4) and those missing baseline scores (*n* = 2; final *n* = 64), are presented in Table 3; Fig. 1. From baseline to the last assessment, both modalities showed increases in SRC scores on average (telehealth: baseline = 45.95, SD = 10.75, range = 16–64; follow-up = 53.36, SD = 10.26, range = 22–68; in-person: baseline = 51.14, SD = 10.38, range = 23–67; follow-up = 55.23, SD = 9.81, range = 35–68). For the pandemic-era telehealth cohort, total SRCS mean scores increased from 45.95 at baseline to 53.89 at Month 3 (SD = 10.62, range = 20–67) and remained similar at Months 6 and 12 (Table 3). For the pre-pandemic in-person cohort, total SRCS mean scores increased from 51.14 at baseline to 53.17 at Month 3 (SD = 9.57, range = 42–68), remained similar at 53.36 at Month 6 (SD = 14.43, range = 21–68), and increased to 55.23 at Month 12 (SD = 9.81, range = 35–68). Subscale scores showed the same general pattern within cohorts (Table 3). Because treatment modality was confounded with pandemic period, these patterns are presented descriptively. Figure 1 displays raw trajectories.

**Table 3:** Descriptive Statistics for Suicide-Related Coping (SRC) Scores Over Time by Treatment Modality (*N* = 64)

			Total SRCS				SRCS-E				SRCS-I		
			*n*	Mean (SD)	Md [Range]		*n*	Mean (SD)	Md [Range]		*n*	Mean (SD)	Md [Range]
**Telehealth**													
	Month 0		43	45.95 (10.75)	48 [16–64]		43	18.56 (4.83)	19 [0–27]		43	20.33 (4.20)	21 [11–28]
	Month 3		35	53.89 (10.62)	56 [20–67]		35	22.66 (4.43)	23 [8–28]		35	22.20 (4.61)	23 [9–28]
	Month 6		35	52.86 (9.84)	53 [35–68]		35	21.86 (4.29)	22 [13–28]		35	23.00 (3.73)	23 [16–28]
	Month 12		33	53.36 (10.26)	54 [22–68]		33	22.06 (4.26)	23 [9–28]		33	22.42 (4.34)	23 [10–28]
**In-person**													
	Month 0		21	51.14 (10.38)	52 [23–67]		21	20.76 (4.56)	21 [12–27]		21	22.95 (3.76)	24 [14.00–28]
	Month 3		12	53.17 (9.57)	51 [42–68]		12	21.25 (4.73)	20 [14–28]		12	23.50 (2.91)	24 [18.00–28]
	Month 6		11	53.36 (14.43)	53 [21–68]		11	21.82 (5.95)	23 [10–28]		11	23.91 (4.39)	24 [15.00–28]
	Month 12		13	55.23 (9.81)	58 [35–68]		13	22.54 (3.43)	23 [15–28]		13	23.69 (3.59)	24 [17.00–28]

SRC = Suicide-Related Coping, SRCS = SRC Scale, SRCS-E = SRCS External Coping subscale, SRCS-I = SRCS Internal Coping subscale, SD = Standard Deviation, Md = Median. Higher SRCS scores indicate better SRC. Month 0 is baseline, Month 3 is the post-treatment assessment, Month 6 is the 3-month post-treatment follow-up, and Month 12 is the 9-month post-treatment follow-up

## Discussion

This study described pre-pandemic onset in-person and pandemic-era telehealth cohorts within the PLF RCT (Goodman et al., 2021, 2026) by examining baseline clinical profiles, treatment engagement patterns, and SRC trajectories across PLF delivery periods. Several descriptive patterns were observed in these naturalistic cohorts.

First, the pandemic-era telehealth cohort showed higher treatment initiation rates (i.e., attending at least one session) than the pre-pandemic in-person cohort (80% and 56.5%, respectively). This pattern is consistent with broader evidence that telehealth can reduce structural barriers to care, such as such as scheduling conflicts, travel distance, transportation costs, and caregiving demands (Cully et al. 2024; Gentry et al. 2018; Hessinger et al. 2026; Holder et al., 2026; Swartz et al. 2023b). Among treatment initiators, engagement rates (≥ 6 sessions: 68.2% and 59.1%) and completion rates (34.1% and 18.2%), as well as total sessions attended (averaging 6.91 and 6.23), were more similar across cohorts than initiation rates. This observation suggests that once initial access barriers are overcome, other treatment factors may sustain participation more than delivery modality. However, because treatment modality was associated with pandemic period, this pattern should be interpreted as descriptive rather than as evidence that telehealth itself caused better treatment initiation.

In this line, the pre-pandemic in-person cohort presented with higher baseline suicidal ideation and SRC than the pandemic-era telehealth cohort (Table 2). These cohort differences may reflect shifts in referral patterns, clinical thresholds for PLF enrollment, or broader contextual changes during the pandemic period, including altered healthcare seeking behaviors and VA suicide prevention practices. Regardless of the reason for these patterns, finding suggest that treatment trials conducted during this period may have enrolled distinct cohorts, which complicates the study of treatment delivery modality during COVID-19 related healthcare changes.

Finally, both cohorts demonstrated overall improvements in SRC over time (Fig. 1). This pattern is consistent with PLF’s intended mechanism of strengthening SSPs through iterative skill practice, which ultimately aims to improve SRC through structured planning (Goodman et al., 2026). Most notably, despite descriptive cohort differences in SRC and suicidal ideation at the beginning of treatment, both treatment modality cohorts demonstrated sustained improvement over the full study period. Given that the transition from in-person to telehealth modality occurred due to the onset of COVID-19 pandemic and caused substantial disruptions to care and daily life, it would have been plausible that the pandemic-era telehealth cohort would show attenuated or less sustained improvement. Instead, descriptive improvements were maintained throughout follow-up, which suggests that PLF’s emphasis on strengthening SRC may be adaptable across both in-person and telehealth delivery contexts. As telehealth can reduce barriers to care, these positive findings lend themselves to the promise of group-based suicide-related interventions delivered via telehealth.

Together, these cohort patterns extend prior suicide-safety planning intervention research and existing PLF telehealth evaluations (Patel et al., 2023; Raciborski et al., 2023) by describing treatment engagement patterns and SRC trajectories in a naturalistic transition from in-person to telehealth delivery. The higher initiation rates in the pandemic-era cohort, alongside sustained SRC increases in both cohorts, are consistent with telehealth being a feasible format for suicide-specific group interventions. In parallel, the potentially distinct cohort characteristics in pre-pandemic and pandemic-era periods indicate that it is important to describe cohort characteristics when delivery modality changes over time.

Future research should examine how cohort-specific characteristics (e.g., suicidal ideation severity, referral source) interact with delivery modality in randomized designs. We did not directly compare modalities because modality transitioned due to disruptions caused by the COVID-19 pandemic and was not randomized by necessity. Therefore, observed naturalistic patterns could reflect a combination of modality effects, pandemic-related factors, temporal and contextual influences, and selection processes (e.g., Rabner et al., 2025). For instance, as noted above, the clinical cohort characterizations suggest potential differences in referral pathways. Relatedly, broader pandemic related changes, including disruptions to social interaction, healthcare access, and daily routines, may have affected treatment engagement and coping strategies measured by the SRCS-17. Randomized modality trials could clarify whether the observed patterns generalize past pandemic contexts and whether specific group processes (e.g., cohesion) function similarly in different treatment modalities.

In sum, these findings add to growing literature describing telehealth delivery for suicide-specific group psychotherapy. Higher treatment initiation in the pandemic-era telehealth cohort, together with SRC improvement in both cohorts, support telehealth as a feasible format for suicide-specific group interventions. Continuing to study how treatment modality relates to treatment engagement and skill acquisition will be essential to guide integration of telehealth group interventions into suicide prevention care.

## Supplementary Information

Below is the link to the electronic supplementary material.

Supplementary Material 1

## Data Availability

Data is not publicly available due to Institutional Review Board regulations and is available on request.

## Declarations

### Author Area of Interest

Intervention development, Suicide prevention, biomarkers.

### Clinical Trial Number

NCT03653637 (registered on ClinicalTrials.gov on 2018-08-31).
