# Supplementary material for: Group-Based Suicide Safety Planning for Veterans with High Suicide Risk Via In-Person and Telehealth Delivery
Source: J Technol Behav Sci. 2026 Sep 18;11(3):645–54. doi: 10.1007/s41347-026-00719-y (PMC13601121; doi:10.1007/s41347-026-00719-y)
Supplement: Supplementary file 1 [file 41347_2026_719_MOESM1_ESM.docx]

**Supplementary Figure 1.** Flow of Participants Through the Project Life Force Randomized Clinical Trial.
